# Sphingosine 1-phosphate lyase ablation disrupts presynaptic architecture and function via an ubiquitin- proteasome mediated mechanism

**DOI:** 10.1038/srep37064

**Published:** 2016-11-24

**Authors:** Daniel N. Mitroi, André U. Deutschmann, Maren Raucamp, Indulekha Karunakaran, Konstantine Glebov, Michael Hans, Jochen Walter, Julie Saba, Markus Gräler, Dan Ehninger, Elena Sopova, Oleg Shupliakov, Dieter Swandulla, Gerhild van Echten-Deckert

**Affiliations:** 1 LIMES Institute, Membrane Biology & Lipid Biochemistry, University of Bonn, Bonn, Germany; 2 Institute of Physiology, University of Bonn, Bonn, Germany; 3 Department of Neurology, Molecular Cell Biology Unit, University of Bonn, Bonn, Germany; 4 Children’s Hospital Oakland Research Institute, University of California, San Francisco, USA; 5 Department of Anaesthesiology and Intensive Care Medicine, Center for Sepsis Control and Care (CSCC), and the Center for Molecular Biomedicine (CMB), University Hospital Jena, Jena, Germany; 6 German Centre for Neurodegenerative Diseases (DZNE), Bonn, Germany; 7 Department of Neuroscience, Karolinska Institutet, 171 77 Stockholm, Sweden; 8 Institute of Translational Biomedicine, St. Petersburg State University, 199034 St. Petersburg, Russia

## Abstract

The bioactive lipid sphingosine 1-phosphate (S1P) is a degradation product of sphingolipids that are particularly abundant in neurons. We have shown previously that neuronal S1P accumulation is toxic leading to ER-stress and an increase in intracellular calcium. To clarify the neuronal function of S1P, we generated brain-specific knockout mouse models in which S1P-lyase (SPL), the enzyme responsible for irreversible S1P cleavage was inactivated. Constitutive ablation of SPL in the brain (SPL^fl/fl/Nes^) but not postnatal neuronal forebrain-restricted SPL deletion (SPL^fl/fl/CaMK^) caused marked accumulation of S1P. Hence, altered presynaptic architecture including a significant decrease in number and density of synaptic vesicles, decreased expression of several presynaptic proteins, and impaired synaptic short term plasticity were observed in hippocampal neurons from SPL^fl/fl/Nes^ mice. Accordingly, these mice displayed cognitive deficits. At the molecular level, an activation of the ubiquitin-proteasome system (UPS) was detected which resulted in a decreased expression of the deubiquitinating enzyme USP14 and several presynaptic proteins. Upon inhibition of proteasomal activity, USP14 levels, expression of presynaptic proteins and synaptic function were restored. These findings identify S1P metabolism as a novel player in modulating synaptic architecture and plasticity.

S1P is an evolutionarily conserved catabolic intermediate of sphingolipid metabolism that has been suggested to regulate crucial functions in the brain including neural development, differentiation and survival^1^,^2^. Its deficiency resulted in embryonic lethality associated with disturbed neurogenesis including neural tube closure^1^. On the other hand its accumulation turned out to be neurotoxic leading to neuronal death^3^,^4^. Alternatively, S1P is proposed as a neuroprotective factor that is lost early in Alzheimer pathogenesis^5^. These controversial findings point to a tight regulation of S1P levels in the brain. However, reports regarding the function of enzymes involved in S1P metabolism are also controversial. There are two isoforms of sphingosine kinases (SK1 and SK2) that generate S1P^6^. Presynaptic SK1-derived S1P was reported to promote neurotransmitter release in hippocampal neurons^7^ and *C. elegans*^8^, while studies in human and rodent brain suggest that SK2 is particularly important in neurons^9^,^10^. However, the major regulator of intracellular S1P levels is S1P-lyase (SPL). It catalyses the irreversible cleavage of S1P to hexadecenal and ethanolamine phosphate, the final step of sphingolipid catabolism^11^.

Others and we have shown that loss of SPL activity results in tissue-dependent accumulation of S1P, sphingosine and other sphingolipid intermediates^3^,^12^,^13^. Intriguingly, SPL-deficiency in neurons causes a predominant S1P accumulation and to a lesser degree, its metabolic precursor sphingosine with no significant alterations in ceramide, sphingomyelin and glycosphingolipids^13^. S1P accumulation in SPL-deficient neurons was associated with increased cytosolic calcium levels^4^ and ER-stress^13^, which mediated apoptotic neuronal death^4^. Based on these findings and due to early postnatal death of systemic SPL knockouts^13^, we assumed that brain-specific ablation of SPL might serve as a tool to clarify the role of neuronal S1P. We therefore generated two mouse models, one with developmental neural-specific, and one with postnatal neuronal forebrain-targeted ablation of SPL and explored the consequences on synaptic plasticity. Our results strongly suggest an involvement of sphingolipid metabolism in maintaining presynaptic nerve terminal architecture and functions leading to cognitive deficits. We further show that S1P accumulation is essential for the assessed elevation of ubiquitin-proteasome system (UPS) which is responsible for the decreased expression of several presynaptic proteins and of the deubiquitinating protease USP14. The latter was shown to play a critical role in synaptic plasticity and its loss is associated with several physiological impairments in the central nervous system^14^.

## Results

### Generation of two tissue-specific SPL knockout mouse models

**Figure 1:**
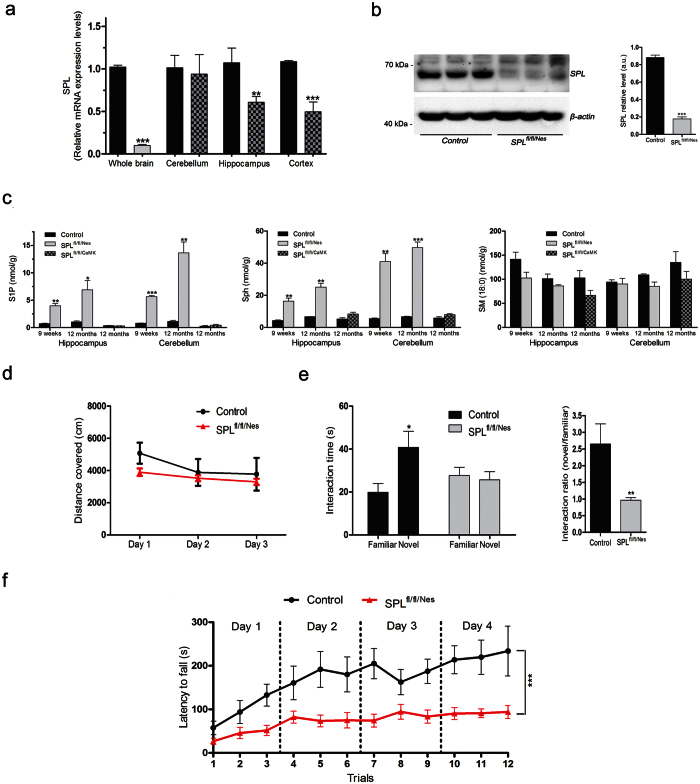
Brain targeted knock-down of SPL and its impact on sphingolipid content and behaviour. SPL expression was assessed in the indicated brain domains of 6 weeks old mice of the indicated genotype by (**a**) qRT-PCR (unpaired t-test, P < 0.0001) (**b**) Western blotting and quantification (unpaired t-test, P < 0.0001) as described in the Methods section (**c**) Sphingolipids from the cerebellum and the hippocampus of mice at the indicated age and genotype were determined by LC/MS/MS as described in the Methods section. Sphingosine 1-phosphate (S1P) and sphingosine (Sph) increased considerably already after 9 weeks in brains of SPL^fl/fl/Nes^ but not SPL^fl/fl/CaMK^ mice. Bars represent means ± SEM (n ≥ 3; two-way ANOVA, P_S1P,h6w _= 0.041, P_S1P,h12m _= 0.0458, P_S1P,c6w _= 0.041, P_S1P,c12m _= 0.0458, P_Sph,h6w _= 0.0326, P_Sph,h12m _= 0.0284, P_Sph,c6w _= 0.0326, P_Sph,c12m _= 0.0284). The amount of all other lipids determined including ceramides did not change upon SPL knockdown (not shown). (**d**) Open field test: exploratory locomotor activity is expressed as the distance covered during 20 min. (**e**), Object placement recognition test: shown is the exploration time of the objects in the novel and familiar location (two-way ANOVA, P = 0.0265) and the discrimination index, represented by the normalized ratio of time spent with the object placed in the novel area and the object in the familiar area (unpaired t-test, P = 0.0097). (**f**) Latency to fall from the accelerating rotarod (two-way ANOVA, P < 0.0001).

Mice in which exons 9/10 and 12/13 encoding for the binding site of the SPL cofactor pyridoxalphosphate (PLP) were flanked by *loxP* sites (*floxed, Sgpl1*^flox/flox^) were crossbred with the nestin-Cre transgenic mouse line Nes-Cre1 in which Cre-recombinase expression is under the control of the nestin promoter^15^. Siblings expressing both, *loxP* sites and Cre-recombinase (SPL^fl/fl/Nes^) exhibited a considerable reduction in the brain on transcriptional and protein level (Fig. 1a,b). The residual mRNA amounting to 10 ± 4% is most probably derived from non-neural cells devoid of an active nestin promoter^15^. Accordingly, a slight protein band was also detectable (Fig. 1b). In contrast to systemic SPL deletion, mice lacking SPL only in neural tissue (SPL^fl/fl/Nes^) exhibit a rather unremarkable phenotype and their lifespan is comparable to that of their wild type littermates, thus representing a promising model to analyze the role of SPL in brain physiology. Post-natal forebrain-specific deletion of SPL was achieved by crossing the floxed SPL mice (*Sgpl*^*flox/flox*^) with a transgenic Cre-expressing line, driven by calcium/calmodulin-dependent protein kinase II α subunit (CaMKII-Cre)^16^. As expected the resulting SPL^fl/fl/CaMK^ mice showed reduced SPL expression only in cortex and hippocampus but not in cerebellum (Fig. 1).

### SPL ablation causes S1P accumulation in brains of SPL^fl/fl/Nes^ but not SPL^fl/fl/CaMK^ mice

Consistent with earlier findings in primary cultured cerebellar neurons generated from mice with systemic SPL deletion^13^, neural-targeted depletion of SPL resulted in a considerable accumulation of S1P and its metabolic precursor sphingosine with no changes in ceramide and sphingomyelin in brains of SPL^fl/fl/Nes^ mice (Fig. 1c). Postnatal neuronal forebrain-restricted ablation of SPL did not cause accumulation of any sphingolipid either in hippocampi (forebrain domain) or cerebella of SPL^fl/fl/CaMK^mice. Obviously, the residual SPL expression in other than CaMKII expressing cells (Fig. 1a) is sufficient to prevent accumulation of S1P in this mouse model. Hence, only SPL^fl/fl/Nes^ mice were investigated further.

### SPL^fl/fl/Nes^ mice exhibit deficits in spatial learning, memory and motor coordination

Based on the neurotoxic effects of S1P reported earlier^4^, we first assessed whether neuronal S1P accumulation affects cognitive skills and motor coordination of SPL^fl/fl/Nes^ mice. First we analysed exploratory activity in an open field and found no significant difference in the distance covered by control and SPL^fl/fl/Nes^ mice indicating that locomotor activity is not affected (Fig. 1d). A cognitive test of object placement recognition showed clear impairments of hippocampal spatial learning and memory in SPL^fl/fl/Nes^ mice (Fig. 1e). In addition, motor coordination and balance with the accelerating rotarod thought to involve cerebellar functions revealed significant impairments in SPL^fl/fl/Nes^mice (Fig. 1f). Thus SPL ablation in neurons is associated with significant behavioural abnormalities. Hence we conducted further experiments in cerebellum and hippocampus. We concentrated on performing physiological and morphological studies mainly in the CA1 region of the hippocampus and biochemical studies in part in cerebellar neurons.

### Altered presynaptic morphology in hippocampal CA1 region of SPL^fl/fl/Nes^ mice

Based on the observed behavioural abnormalities and our previous results on S1P neurotoxicity^4^, we tested whether brains of SPL^fl/fl/Nes^ mice exhibit neuronal loss. In a first straight forward approach a neuron-specific nuclear protein (NeuN) immunohistochemical staining of coronal brain sections was performed. Although we did not observe massive neuronal loss, closer quantitative examination of brain sections revealed a reduced thickness of the dentate gyrus in SPL^fl/fl/Nes^mice (suppl. Fig. 1). These gross irregularities in the hippocampal region prompted us to analyse the hippocampal morphology in more detail.

We first assessed the subcellular morphology of asymmetric (excitatory) synapses in the hippocampal CA1 region in SPL^fl/fl/Nes^mice.

**Figure 2:**
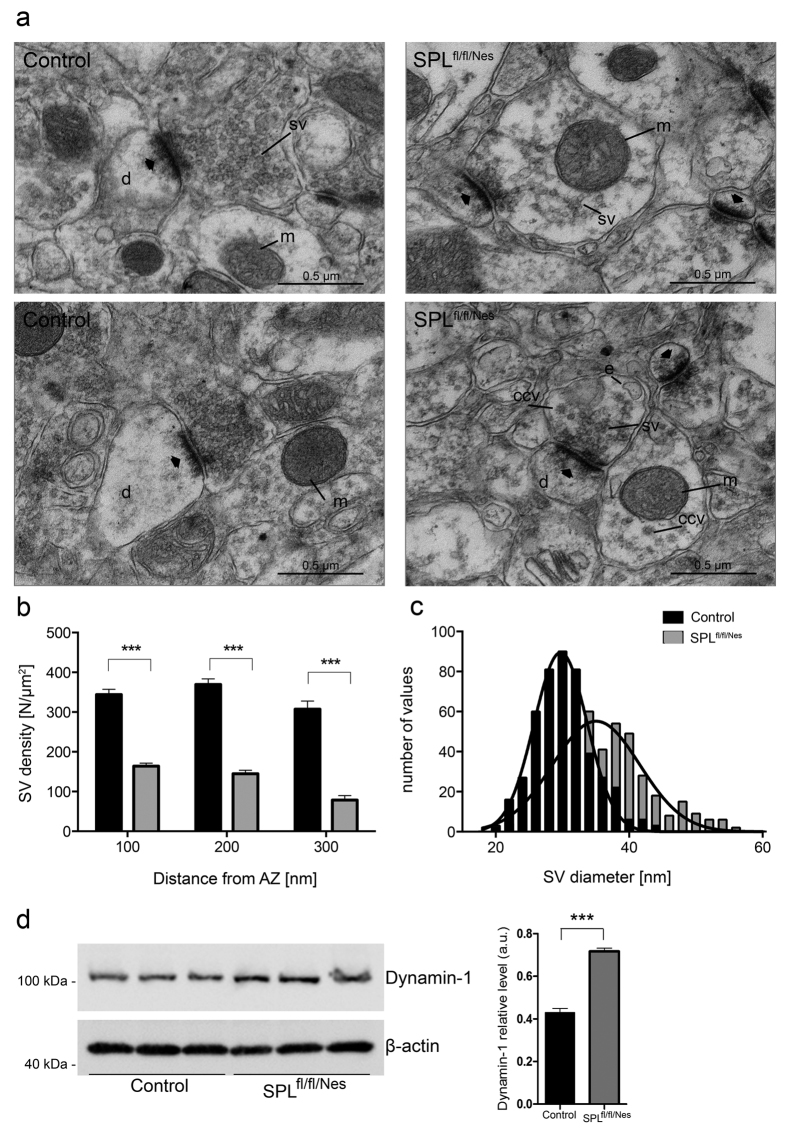
Subcellular morphology of asymmetrical synapses in CA1 region of murine hippocampus following ablation of SPL. Representative electron micrographs of hippocampal synapses from controls (n = 3) and SPL^fl/fl/Nes^mice (n = 3) **(a**). sv, synaptic vesicles, d, dendritic spines, e, endosomal profile, ccv, clathrin coated vesicle, m, mitochondrion. Thick arrows indicate synaptic contacts. (**b)** bar graph showing average SV density within 100, 200, and 300 nm regions from the border of the active zone; (n = 62 synapses from 3 animals; ***P < 0.0001, two-tailed t-test). (**c**) frequency distribution of SV diameters from CA1 presynaptic boutons from control and SPL^fl/fl/Nes^mice, mean ± SEM, 36.0 ± 0.3 vs 30.3 ± 0.2, n = 460; P < 0.0001, two-tailed t-test). Solid lines represent Gaussian fits for each distribution. (**d**) expression of dynamin-1 in hippocampal lysates of control and SPL^fl/fl/Nes^mice (P = 0.0003, two-tailed t-test).

Analysis of ultrathin sections revealed a significant decrease in number and density of synaptic vesicles in nerve terminals from SPL^fl/fl/Nes^mice compared to controls (Fig. 2a). Density of synaptic vesicles (SVs) localized in the area 300 nm from the border of the active zone was decreased in SPL^fl/fl/Nes^mice compared to controls (controls: 340.3 ± 12.3; SPL^fl/fl/Nes^mice: 129.1 ± 7.3 N/μm^2^; P < 0.0001) (Fig. 2b). The reduction was also evident in each of 100 nm steps from the border of the active zone, indicating that the number of SVs is reduced not only in the reserve pool, but also in the pool proximal to the active zone. Unexpectedly, the number of vesicles associated with the presynaptic membrane per μm^2^ of the active zone at rest was not reduced significantly (controls: 115.4 ± 48.2 and SPL^fl/fl/Nes^mice: 98.5 ± 38.0; P = 0.345; n = 28 synapses; two-tailed t-test). This result suggests that once available SVs can be efficiently recruited to sites of release. Further, the diameter of SVs in SPL^fl/fl/Nes^mice was significantly larger compared to controls (Fig. 2c). Similar findings were observed for mossy fiber synapses in CA3 region (suppl. Fig. 2). The number of clathrin-coated endocytic intermediates was significantly increased in nerve terminals with ablated SPL as compared to controls (controls: 0.13 ± 0.09 and SPL^fl/fl/Nes^mice: 1.77 ± 0.35 and; P < 0.001; n = 62 synapses; two-tailed t-test). An increased expression of dynamin was also observed in SPL^fl/fl/Nes^mice (Fig. 2d). No significant difference in the length of the active zone was found in synapses from SPL^fl/fl/Nes^ and controls (0.23 ± 0.01 and 0.24 ± 0.01, P = 0.224; n = 62; two-tailed t-test).

### Spontaneous mEPSCs are altered in SPL^fl/fl/Nes^mice

**Figure 3:**
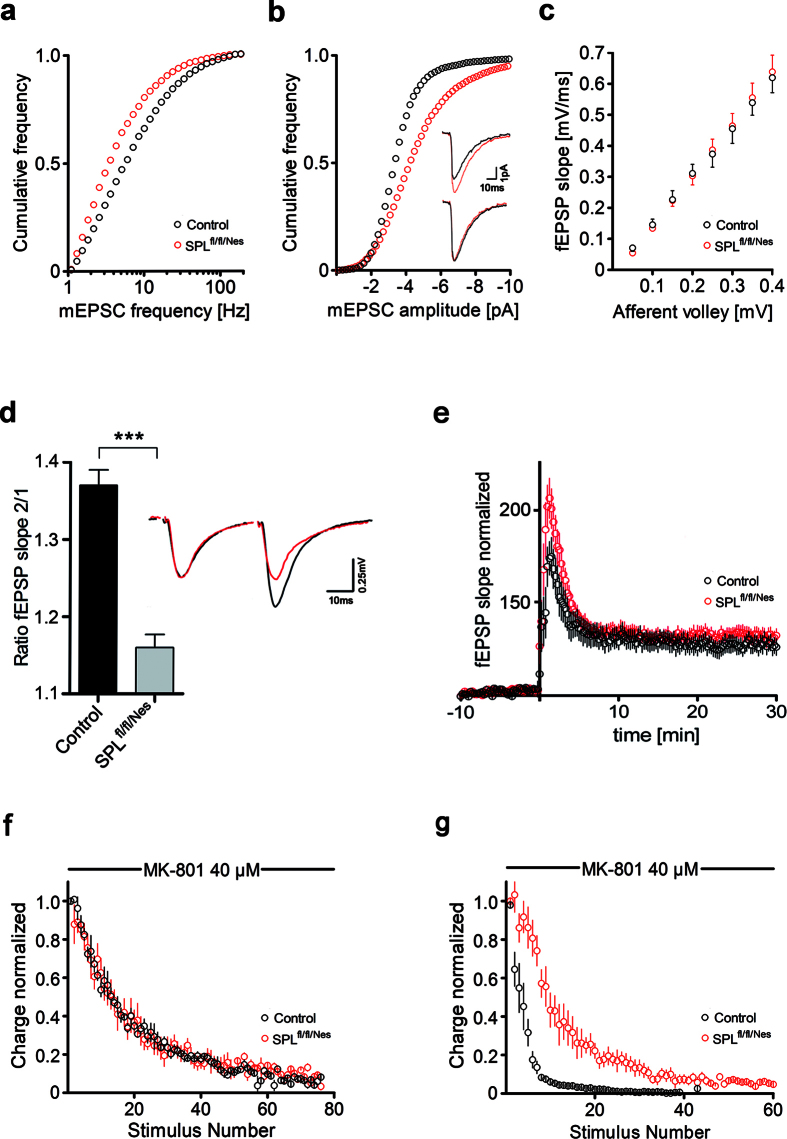
Electrophysiology of Schaffer collateral synapses in CA1 region of murine hippocampus with ablation of SPL. (**a**) mEPSC frequency is significantly reduced and (**b**) mEPSC amplitude significantly increased in SPL^fl/fl/Nes^slices compared to controls. Cumulative frequencies of occurrence of mEPSC frequencies and mEPSC amplitudes are plotted. (**b**) Inset shows examples of mEPSC traces (black: control; red: SPL^fl/fl/Nes^), lower traces are normalized in amplitude. (**c)** normal basal synaptic transmission as shown in the input-output curves. (**d)** PPF is significantly reduced at Schaffer collateral synapses from SPL^fl/fl/Nes^ mice compared to controls. Ratios of the initial slopes of fEPSP pairs are plotted. Inset shows example traces at 40 ms stimulus interval (black: control; red: SPL^fl/fl/Nes^). (**e)** LTP recorded in the hippocampal CA1 region after high frequency stimulation (100 Hz, 1 s) of Schaffer collaterals in control and SPL^fl/fl/Nes^ slices is unchanged. fEPSP slopes are expressed as percentage of change from baseline recordings (taken to be 100%) and plotted against time. (**f**) blocking rate of the NMDA responses by 40 μM MK-801 does not significantly vary between control and SPL^fl/fl/Nes^ synapses. Results are displayed as 3 point bins. (**g)** blocking rate of the NMDA responses elicited by short trains (3 pulses, 10 ms interval, 0.05 Hz) is slower at SPL^fl/fl/Nes^ synapses compared to controls. Results are displayed as 3 point bins. (**a,b**) n: mEPSCs/mice, control: 4038/6, SPL^fl/fl/Nes^: 3886/6; data in c-g represent means ± SEM; **c**, n: slices/mice, control: 12/3, SPL^fl/fl/Nes^: 11/3; (**d**) n: slices/mice, control: 30/10, SPL^fl/fl/Nes^: 48/16; **e**, n: slices/mice, control: 15/5, SPL^fl/fl/Nes^: 19/6; (**f**) n: neurons/mice, control: 5/4, SPL^fl/fl/Nes^: 5/4; (**g**) n: neurons/mice, control: 6/4, SPL^fl/fl/Nes^: 6/5; unpaired t-test, ***P < 0.0001.

To determine whether the changes in vesicle size and number were also reflected in the properties of the spontaneous release, we measured mEPSCs in pyramidal cells in the CA1 region. The frequency of the spontaneous events in SPL^fl/fl/Nes^mice (Fig. 3a) was significantly reduced (P < 0.001) while the current amplitude (Fig. 3b) was significantly increased (P < 0.001). The latter finding is in line with the observation of larger synaptic vesicles in SPL^fl/fl/Nes^mice (Fig. 2c). Current kinetics of mEPSCs were unchanged in SPL^fl/fl/Nes^mice compared to controls (Fig. 3b inset).

### SPL^fl/fl/Nes^mice exhibit a reduction in paired pulse facilitation (PPF)

Based on the observed alterations in synaptic morphology of SPL^fl/fl/Nes^mice, we next assessed the physiological consequences of S1P accumulation on synaptic plasticity in hippocampal CA1 region. Dendritic field potential recordings in acute hippocampal slices from SPL^fl/fl/Nes^mice revealed normal basal synaptic transmission (Fig. 3c), but exhibited a significant reduction in PPF at Schaffer collateral synapses between 2 to 12 months of age (Fig. 3d). In contrast, induced long-term potentiation (LTP) was not altered in SPL^fl/fl/Nes^mice within this age range (Fig. 3e). Because the amount of PPF is normally inversely related to initial release probability^17^,^18^ the possibility exists that the decrease in PPF in SPL^fl/fl/Nes^mice is derived from an increase in initial release probability. To assess initial release probability we measured the rate of block of NMDA receptors by the use-dependent blocker MK-801^19^. An increase in the release probability would cause an increase in the rate of block. No differences in the rate of block by 40 μM MK-801 were observed using single pulse stimulation in SPL^fl/fl/Nes^ mice compared to control animals (Fig. 3f). The initial release probability in SPL^fl/fl/Nes^mice therefore seems to be unaltered. Using facilitated pulses (3 pulses, 100 Hz) the rate of block by MK-801 was reduced by ~300% (Fig. 3g), indicating a lower release probability of facilitated responses. This suggests that the observed decrease in PPF is due to a reduction in the release probability of the second pulse in SPL^fl/fl/Nes^ mice compared to control mice.

### Altered expression of presynaptic proteins in SPL^fl/fl/Nes^mice

**Figure 4:**
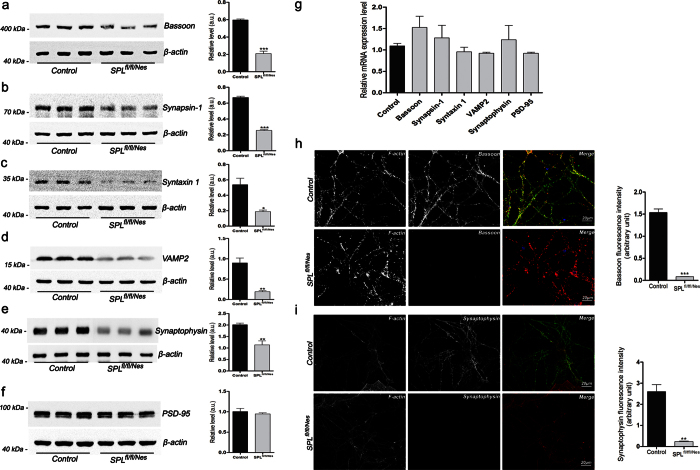
Expression of presynaptic proteins is reduced in SPL^fl/fl/Nes^ mice. (**a–f**) Protein amounts were assessed by immunoblotting (unpaired t-test, P_Bassoon_ = 0.0002, P_Synapsin-1_ < 0.0001, P_syntaxin 1_ = 0.0157, P_VAMP2_ = 0.0048, P_Synaptophysin_ = 0.0099), and (**g**) transcript amounts were determined by qRT-PCR in hippocampi of 6-month-old mice. Immunostaining of the presynaptic marker proteins (**h**) Bassoon and (**i**) synaptophysin in cerebellar granule cells after 2 weeks in culture (unpaired t-test, P_Bassoon_ < 0.0001, P_Synaptophysin_ = 0.0034). F-actin (red), bassoon and synaptophysin (green).

Next, we aimed to identify the molecular mechanism underlying the observed alterations in synaptic morphology and function in SPL-deficient brains. First we assessed whether the reduced number of synaptic vesicles is also reflected in the expression of presynaptic proteins. We found that in hippocampi as well as in cultured cerebellar neurons of SPL^fl/fl/Nes^mice the expression of the presynaptic markers Bassoon and Synapsin-1 as well as the SNARE-proteins syntaxin and VAMP2 and the major synaptic vesicle protein synaptophysin was significantly decreased (Fig. 4a–e). However, the expression of other presynaptic proteins including synaptotagmin 1, piccolo, SNAP25, Munc18 and NCS-1 was not affected (Suppl. Fig. 4a–e). The expression of the post-synaptic marker PSD-95 (Fig. 4f) and other proteins including IDE (insulin degrading enzyme) and GAP-43 (Suppl. Fig. 4f,g) was not affected in SPL^fl/fl/Nes^mice. Since S1P was shown to modulate the activity of histone deacetylases^20^, we checked whether the decreased expression of presynaptic proteins occurred at transcriptional level. Yet, no changes in transcript amounts were found in brains of SPL^fl/fl/Nes^mice (Fig. 4g). In cultured cerebellar neurons of SPL^fl/fl/Nes^mice, reduction in presynaptic marker proteins Bassoon and synaptophysin were even more pronounced (Fig. 4h,i).

### The ubiquitin-proteasomal system is up-regulated in SPL^fl/fl/Nes^mice

**Figure 5:**
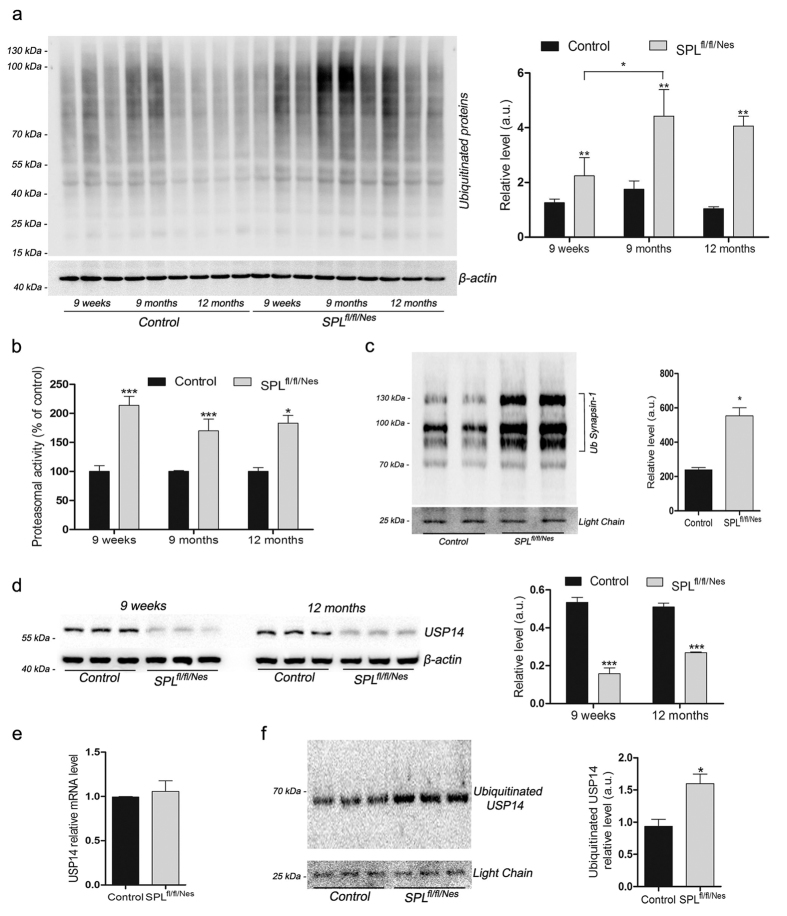
Ablation of SPL leads to an elevation of UPS activity and a decreased expression of USP14. Experiments were performed in brains of SPL^fl/fl/Nes^ mice at the indicated ages. (**a**) Age-dependent increase of ubiquitinated proteins (two-way ANOVA, P_genotype _= 0.0058, P_time _= 0.0284). (**b**) increase of proteasomal activity (two-way ANOVA, P_genotype _= 0.0058). (**d**) expression of USP14 (two-way ANOVA, P_genotype _< 0.0001).(**e**) transcript levels of USP14. (**c,f**) immunoprecipitation of ubiquitinated (Ub) proteins followed by Western blotting of synapsin-1 and USP14 (unpaired t-test, P_Synapsin-1_ = 0.0239, P_USP14_ = 0.0231).

As many presynaptic proteins are known to be degraded by the UPS^21^,^22^, we next assessed protein ubiquitination and proteasomal activity in SPL^fl/fl/Nes^ brains. The amount of ubiquitinated proteins was indeed considerably elevated in brains of SPL^fl/fl/Nes^mice compared to the respective age matched controls (Fig. 5a). Moreover, augmented protein ubiquitination was associated with increased proteasomal activity (Fig. 5b). In addition we showed that the presynaptic protein synapsin, under-expressed in SPL^fl/fl/Nes^ mice was indeed included in the ubiquitinated protein fraction (Fig. 5c). Surprisingly, we obtained 3 clear-cut bands and not the typical ubiquitin smear indicating that three different ubiquitinated species of synapsin (corresponding most likely to one, two and six ubiquitin units) are predominant in this fraction.

The proteasome-associated deubiquitinating enzyme USP14 on one hand is functionally coupled with proteasomal activity and on the other hand a critical regulator of synaptic plasticity^14^. We therefore studied the expression of USP14 at protein and mRNA level in SPL^fl/fl/Nes^mice. As depicted in Fig. 5d the expression of USP14 was considerably reduced in hippocampi of SPL^fl/fl/Nes^mice. However, there were no significant differences in the amount of the respective transcripts (Fig. 5e). On the other hand USP14 was included in the highly ubiquitinated protein fraction, suggesting that its decreased expression could be due to its degradation by the proteasome (Fig. 5f).

### Proteasome inhibition restores PPF as well as expression of USP14 and calcium chelation normalizes proteasomal activity

**Figure 6:**
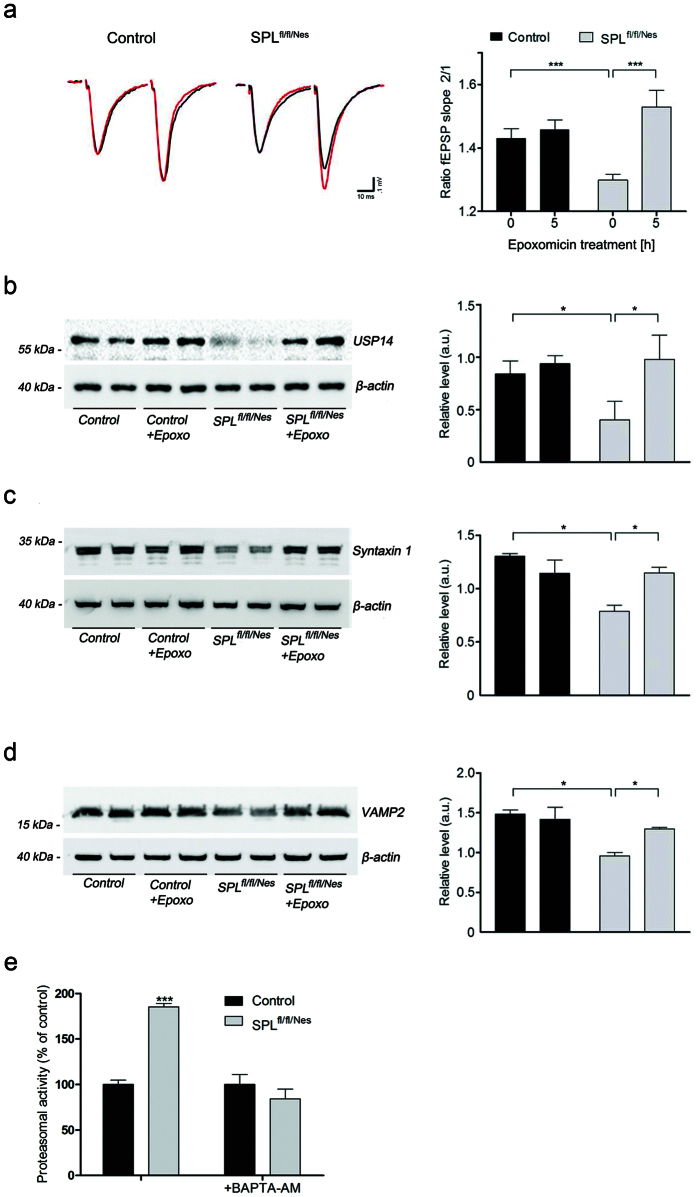
Proteasome inhibition restores paired-pulse facilitation (PPF), USP14 as well as SNARE- proteins and calcium chelation normalizes proteasomal activity. Hippocampal slices from control and SPL^fl/fl/Nes^ mice were incubated in the absence (black traces) or presence (red traces) of 15 μM epoxomicin (Epoxo) for 5 h. (**a**) PPF n: slices/mice, control: 15/6, SPL^fl/fl/Nes^: 16/7 (two-way ANOVA, P_genotype _= 0.0009, P_Epoxo _= 0.0003). (**b**–**d**) immunoblotting of USP14 and SNARE-proteins (two-way ANOVA, P_USP14,genotype _= 0.0125, P_USP14,Epoxo _= 0.0251, P_Syntaxin 1,genotype _= 0.0315, P_Syntaxin 1,Epoxo _= 0.0138, P_VAMP2, genotype _= 0.0390, P_VAMP2,Epoxo _= 0.0426). (**e**) Neurons were incubated in the presence of the calcium chelator BAPTA-AM (5 μM, 1 h) and proteasomal activity was assessed (two-way ANOVA, P_ _= 0.0003).

To test whether slowing protein degradation would restore PPF in the hippocampus of SPL^fl/fl/Nes^mice we treated the hippocampal slices with the proteasome-specific inhibitor epoxomicin for 5 h, restoring PPF at Schaffer collateral synapses in SPL^fl/fl/Nes^mice with no significant effect in controls (Fig. 6a). Similarly, the decreased expression of USP14 and the SNARE-proteins syntaxin1 and VAMP2 was restored upon treatment with epoxomicin (Fig. 6b–d). Restoration of presynaptic proteins was also recapitulated in cultured cerebellar neurons from SPL^fl/fl/Nes^mice (suppl. Fig. 3a,b). As calcium is a potential inducer of the UPS^23^, and we have shown earlier that S1P accumulation induces calcium release from the ER, the effect of calcium chelation on UPS was assessed. Indeed addition of BAPTA-AM completely re-established proteasomal activity in neurons derived from SPL^fl/fl/Nes^ mice (Fig. 6e).

## Discussion

Here we show that neural-targeted ablation of SPL and the consequent accumulation of S1P and sphingosine leads to behavioural, morphological, physiological and molecular abnormalities. Precisely, alterations in presynaptic architecture and short term plasticity were accompanied by a calcium mediated elevation of the UPS and hence reduced expression of several synaptic proteins. The inhibition of proteasomal activity restoring both synaptic plasticity and protein expression suggests the UPS as a possible link connecting sphingolipid metabolism and presynaptic pathology.

Synaptic pathology has been acknowledged as a key early event in neurodegeneration, and presynaptic terminal changes during ageing and neurodegeneration have been reported^24^,^25^. However, the detailed mechanisms connecting sphingolipid metabolism to synaptic dysfunction remain poorly understood and the existing reports on the connection between S1P and sphingosine and synaptic function are rather contradictory^8^,^26^,^27^,^28^,^29^. Earlier reports argue for a positive role of S1P in synaptic transmission^8^,^27^,^28^,^29^. Kanno *et al*.^29^ have shown that exogenously applied S1P to hippocampal slices led to an increase in the rate of mEPSCs and further demonstrated that SK1 knockout mice had impaired LTP along with poor performance in Morris water maze test. In contrast, a recent report demonstrated a repressive effect of S1P signalling on synaptic plasticity^26^. Consistently, our results argue in favour of S1P and sphingosine accumulation leading to perturbations in synaptic morphology and function. Although no changes in LTP were evident our mice exhibited significant learning deficits. Obviously, like shown in earlier studies defects in short term plasticity are sufficient to cause profound impairments in learning^30^,^31^. Hence S1P can be viewed as a double edged sword wherein, despite its importance in normal cellular functions, both decrease and increase of the lipid beyond a threshold might affect cellular functions.

The alterations in synaptic transmission observed in the present study were accompanied by morphological changes of hippocampal synapses. Vesicle pools were largely reduced (60%) and single vesicles were increased in diameter. Consistent with the latter findings, mEPSCs in SPL^fl/fl/Nes^ mice were increased in amplitude and occurred at lower frequency compared to control mice. Analysis of mEPSCs revealed no differences in kinetics for current activation and decay between control and SPL^fl/fl/Nes^ animals suggesting no change in postsynaptic AMPA receptors. Similarly, decay kinetics of evoked postsynaptic currents (eEPSCs) were identical between the first and last eEPSC within a high-frequency stimulus train (data not shown) suggesting that NMDA receptors were also not affected. This is supported by the findings from experiments on initial release probability where NMDA receptor mediated EPSCs were unaltered in SPL^fl/fl/Nes^mice compared to controls. However, we cannot rule out the possibility that changes in the properties of postsynaptic receptors might contribute to the observed differences in spontaneous mEPSCs.

Normally, a reduction in PPF is accompanied by an increase in initial release probability^17^,^18^. However, there are also examples of changes in PPF that are not caused by changes in the initial release probability^32^,^33^,^34^,^35^. In the study by Walters *et al*.^35^ a reduction in the deubiquitinating enzyme USP14 impairs PPF without changing the initial release probability. Instead the loss of USP14 led to a large reduction in the number of presynaptic vesicles. Such a mechanism might also contribute to the synaptic changes observed in SPL^fl/fl/Nes^mice. This is supported by our finding that PPF in SPL^fl/fl/Nes^mice was restored to control levels after inhibition of USP14 protein degradation.

Note that based on the known effects of increased calcium levels on endocytosis^36^,^37^ an increased recycling and delivery of vesicles to release sites may be expected. In agreement with this, no difference in the number of “docked” vesicles was detected in synapses at rest. Moreover, an increased level of dynamin-1 and an increased number of coated endocytic vesicles were observed in the SPL-deficient brains. Additional effects of endocytosis and calcium on the increased size of synaptic vesicles in hippocampi of SPL^fl/fl/Nes^mice remain unclear.

Deletion studies have helped to identify presynaptic proteins which are essential for synaptic function and integrity. Removal of such key proteins often leads to impaired neurotransmitter release, changes of the vesicle pools or cytomatrix at the active zone. However, in some cases the loss of function can be compensated by other presynaptic proteins^38^,^39^,^40^. On analyzing the levels of selected key proteins involved in different stages of the vesicle cycle, we found that several of these proteins were reduced in SPL^fl/fl/Nes^mice. Since we neither observed a change in synaptic strength nor morphological changes in the active zone we conclude that the observed reductions in presynaptic proteins do not underlie or at least are not sufficient^41^ to cause the observed changes in synaptic transmission in SPL^fl/fl/Nes^mice. However, at the structural level a reduction in number of synaptic vesicles in the pool has been reported in synapsin-1 and synapsin triple-knockout mice^42^,^43^. Accordingly, a profound reduction in number of vesicles has been reported also in the current study. A redistribution of synapsin-1 has been recently reported following application of S1P in nanomolar concentration to synapses^44^ thus suggesting that this protein might be a direct target of S1P action. On the other hand, restoration of proteasomal activity and hence expression of USP14 and presynaptic proteins rescued PPF in SPL^fl/fl/Nes^mice.

**Figure 7:**
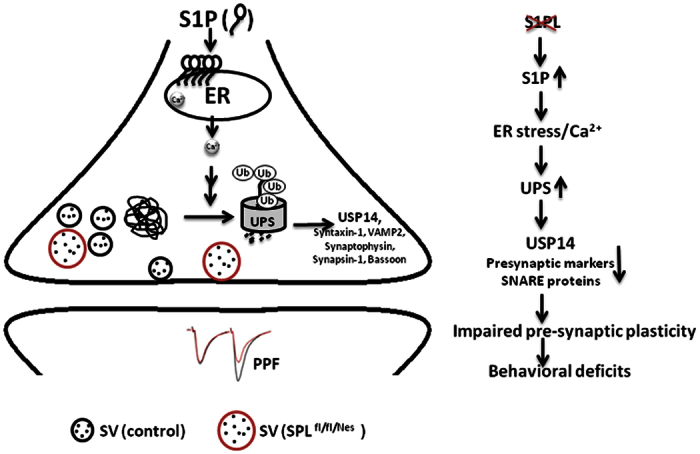
Schematic of proposed molecular mechanism linking SPL activity, synaptic transmission and cognitive skills. S1P accumulation upon SPL deletion causes elevation of cytosolic Ca^2+^, which in turn increases the activity of the ubiquitin-proteasomal system (UPS). As a consequence the expression of the proteasome-associated deubiquitinating enzyme USP14 and of some presynaptic proteins is reduced, thereby impairing presynaptic plasticity coupled to behavioural and motor coordination deficits. PPF, paired pulse facilitation; SV, synaptic vesicle.

In line with our findings in SPL^fl/fl/Nes^ mice, USP14 was shown to regulate hippocampal short-term plasticity and vesicle number^35^. In addition to regulatory effects of USP14 on synaptic activity in ataxia mice^45^ a protective role for the same against Huntington’s cell aggregates has been implicated^46^. We propose the degradation of USP14 by UPS as the underlying molecular mechanism responsible for the physiological and behavioural impairments observed (Fig. 7). Localised in the 19 S regulatory subunit of the proteasome, USP14 has been proposed as a negative modulator of proteasome-mediated degradation^47^. Nevertheless we are aware of the complexity of USP14 functions in the CNS^48^. The substrate specificity of USP14 has been documented before^47^. In line with this we also observed reduced expression of some presynaptic proteins, whereas other proteins were unchanged. The increased expression of dynamin-1 might be explained by other mechanisms like its direct interaction with a chaperone complex reported to stabilize its level at the synapse^49^.

Thus our results indicate a crucial role of proteasomal activity and hence deregulated protein degradation in altered synaptic transmission induced by SPL deficiency. Although, we cannot conclusively pinpoint the exact underlying molecular mechanism, our results, which are in line with recent findings of Walters *et al*.^35^, suggest that quenching of USP14 by the elevated UPS activity might be the central switch that propagates the observed synaptic pathology in SPL^fl/fl/Nes^mice. Since presynaptic dysfunction might be an early pathogenic event in neurodegeneration^50^, our findings could have important implications for diseases where S1P analogues are used as disease modifying therapies.

## Methods

### Mice

All animal experiments were conducted in accordance with the guidelines of the Animal Care Committee of the University of Bonn (LANUV NRW, Az. 87-51.04.2011.A049).

The *Sgpl1*^*flox/flox*^ lines were generated as recently described^51^. *Sgpl1*^*flox/flox*^ mice, harbouring “floxed” exons 10–12 on both *Sgpl1* alleles were crossbred either with mice expressing *nestin-Cre* transgene or the calcium/calmodulin-dependent protein kinase II α subunit-Cre transgene (*CaMKII-Cre*). Thus SPL^fl/fl/Nes^ and SPL^fl/fl/CaMK^ mice in which “floxed” exons are excised by *Cre* recombinase were obtained.

### Ethical statement

All animal experiments were conducted in accordance with the guidelines of the Animal Care Committee of the University of Bonn. The experimental protocols were approved by Landesamt für Natur, Umwelt und Verbraucherschutz Nordrhein-Westfalen (LANUV) (LANUV NRW, Az. 87-51.04.2011.A049).

### Antibodies

Monoclonal antibodies against synapsin-1, synaptophysin, PSD95, Bassoon, SNAP25, VAMP2 and ß-Actin (8H10D10), anti synaptotagmin1 polyclonal antibody, secondary antibodies including HRP-linked anti-rabbit and anti-mouse IgG, and fluorescent secondary antibodies (anti-rabbit IgG (H+L), F(ab’)_2_ Fragment-Alexa Fluor 488 conjugated and anti-mouse IgG (H+L), F(ab’)_2_ Fragment-Alexa Fluor 555 conjugated) were from Cell Signaling Technology (Cambridge, UK). Anti-piccolo polyclonal serum was from Synaptic Systems (Göttingen, Germany), anti-syntaxin1a polyclonal antibody from Abcam (Cambridge, UK), anti-ubiquitinylated proteins, clone FK2 (mouse monoclonal IgG1) from Millipore (Darmstadt, Germany), rabbit polyclonal anti USP14 was from Thermo Fisher (Rockford, IL, USA).

### Cell culture

Granular cells were cultured from the cerebella of 6-day-old mice as previously described^52^. Briefly, neurons were isolated by mild trypsinization (0.05%, w/v) and dissociated by passing them repeatedly through a constricted Pasteur pipette in a DNase solution (0.1%, w/v). The cells were then suspended in Dulbecco’s Modified Eagle’s Medium containing 10% heat-inactivated horse serum supplemented with 100 units/ml penicillin and 100 mg/ml streptomycin and plated onto 15 mm sterile glass coverslips placed in 6-well plates, 35 mm in diameter, and precoated overnight at 37 °C with 0.01 mg/ml of Poly-L-Lysin dissolved in 1 x PBS (5 × 10^5^ cells/well). After 10 days in culture cells were used for experiments as indicated.

### Lipid extraction and quantification

Lipid measurements were performed according to an established protocol using liquid chromatography coupled to triple-quadrupole mass spectrometry (LC/MS/MS)^53^. Tissue samples were homogenized using the Stomacher Model 80 MicroBiomaster Blender (Seward) in 5 ml PBS after addition of C17-base sphingosine (Sph), C17-base S1P, C17-base sphingomyelin, and C15-base ceramide (Cer) as internal standards (300 pmol/sample, Avanti Polar Lipids). One ml supernatants were transferred into glass centrifuge tubes and mixed with 200 μl of 6 N hydrochloric acid and 1 ml methanol, and vigorously vortexed for 5 min in the presence of 2 ml chloroform. Aqueous and chloroform phases were separated by centrifugation for 3 min at 1900× *g*, and the lower chloroform phase was transferred into a new glass centrifuge tube. After a second round of lipid extraction with additional 2 ml chloroform, the two chloroform phases were combined and vacuum-dried at 50 °C for 50 min using a vacuum concentrator. The extracted lipids were dissolved in 100 μl methanol/chloroform (4:1, v/v) and stored at −20 °C. Detection was performed with the QTrap triple-quadrupole mass spectrometer (ABSciex, Concord, Canada) interfaced with the 1100 series chromatograph (Agilent, Santa Clara, California, USA), the Hitachi Elite LaChrom column oven (VWR, Radnor, Pennsylvania, USA), and the Spectra System AS3500 autosampler (Thermo Separation Products). Positive electrospray ionization (ESI) LC/MS/MS analysis was used for detection of sphingosine 1-phosphate (S1P), Sph, and sphingomyelin (SM), positive atmospheric pressure chemical ionization (APCI) for detection of Cer, hexosylcerebrosides (GalCerBr), and cholesterol (Chol). Multiple reaction monitoring (MRM) transitions were as follows: S1P m/z 380/264, C17-S1P m/z 366/250, Sph m/z 300/282, C17-Sph m/z 286/268, C15-Cer m/z 524/264 (positive mode) 522/266 (negative mode), C16-Cer m/z 538/264, C18-Cer m/z 566/264, CerBr (24:1) m/z 810/264, SM (16:0) m/z 703/184, SM (18:0) m/z 731/184, SM (17:0) m/z 717/184, Chol m/z 369/161. Liquid chromatographic resolution of all analytes was achieved using a 2 × 60 mm MultoHigh C18 reversed phase column with 3 μm particle size (CS-Chromatography Service). The column was equilibrated with 10% methanol and 90% of 1% formic acid in H_2_O for 5 min, followed by sample injection and 15 min elution with 100% methanol with a flow rate of 300 μl/min. Standard curves were generated by adding increasing concentrations of the analytes to 300 pmol of the internal standard. Linearity of the standard curves and correlation coefficients were obtained by linear regression analyzes. Data analyzes were performed using Analyst 1.6 (ABSciex, Concord, Canada).

### Reverse transcription and real-time PCR

Total RNA was extracted from brains using RNeasy Mini Kit (QIAGEN, Hilden, Germany), and treated with RNase-free DNase (QIAGEN) according to the manufacturer’s instructions. Reverse transcription of 1 μg of total RNA was performed using the First Strand cDNA Synthesis Kit (ProtoScript II, New England BioLabs, Frankfurt am Main, Germany). The primers for real-time PCR were designed using the online tool from Life Technologies „Custom Primers - OligoPerfect™ Designer” and obtained from the same company. They are listed as follows: name: forward primer, reverse primer: bassoon: 5′TACACCGCTCTTCCTGCTCT3′, 5′TGTACTCGCTGCCAGACTTG3′; synapsin-1: 5′TCCAGAAGATTGGGCAGAAC3′, 5′TCAGACATGGCAATCTGCTC3′; synaptophysin: 5′AGTACCCATTCAGGCTGCAC3′, 5′CCGAGGAGGAGTAGTCACCA3′; syntaxin 1: 5′GAACAAAGTTCGCTCCAAGC3′, 5′ATTCCTCACTGGTCGTGGTC3′; vamp2: 5′TGACGGTTCCCATCACCTCTC3′, 5′CTGTGGGGTTTGCTTTTGTT3′; psd-95: 5′TTTCTCCCACACACATTCCA3′, 5′ACCTTCCACTCATGCAAACC3′; s1p-lyase: 5′TTTCCTCATGGTGTGATGGA3′, 5′CCCCAGACAAGCATCCAC3′; β-actin: 5′CCACAGCTGAGAGGGAAATC3′, 5′TCTCCAGGGAGGAAGAGGAT3′; 12.5 μl of Power SyBR Green (Applied Biosystems, Carlsbad, California, USA), 2 μl of each primer, 1.5 μl of cDNA, and 9 μl of H_2_O were loaded into a 96 well plate and PCR performed in a 7300 Real Time PCR System (Applied Biosystems, Carlsbad, California, USA). Results were calculated using the relative C_T_ method. The fold increase or decrease was determined relative to controls after normalizing to β-actin as housekeeping gene.

### Western blotting and immunoprecipitation

Total brains, hippocampi or cultured neurons were homogenized twice for 2 min using metallic beads at a frequency of 20 Hz in RIPA buffer (20 mM Tris-HCl, pH 7.5, 150 mM NaCl, 1 mM EDTA, 1 mM EGTA, 1% NP-40, 1% NaDC, 2.5 mM Na_4_ P_2_O_7_, 1 mM b-glycerophosphate, 1 mM Na_3_VO_4_, 1 μg/ml leupeptin). Samples were kept on ice for 1 h followed by centrifugation at 13,000 rpm at 4 °C for 1 h. Proteins were immunoprecipitated from cleared lysates, containing protease inhibitor cocktail (AMRESCO, OH, USA), using anti-ubiquitinylated proteins antibody (5 μg/ml) (clone FK2, mouse monoclonal IgG1, from Millipore, Darmstadt, Germany) for 4 hrs at 4 °C followed by incubation with protein G-conjugated sepharose (GE Healthcare, Little Chalfont, UK) for 1 h at 4 °C. Precipitates were rinsed three times with washing buffer (50 mM Tris/HCl, pH 7.4, 500 mM NaCl, 2 mM EDTA, 0.2% Igepal) for 10 min at 4 °C. Precipitates were collected by centrifugation (4,000× g, 4 °C, 5 min) and eluted by incubation with SDS sample buffer (25 mM Tris/HCl, pH 6,8, 10% Glycerin, 1,5% SDS, 20 mM DTT) for 10 min at 95 °C. Samples were separated by SDS-PAGE and transferred on nitrocellulose membrane. The membrane was incubated with anti-synapsin-1 or anti-USP14 primary antibody overnight at 4 °C. Lysates from total brain, hippocampus and cell cultures were also incubated with SDS sample buffer for 10 min at 95 °C. Proteins were separated by SDS-PAGE, images were cropped and quantified as described earlier^4^.

### Immunocytochemistry

Coverslips with cerebellar neurons were washed with phosphate-buffered saline (PBS) and then fixed with 4% paraformaldehyde for 10 min. Then cells were permeabilized with 0.25% Triton-X100-PBS for another 10 min and blocked with 5% BSA in 0.125% Triton X-100 in PBS for 1 h. After blocking cells were incubated for 1 h with the primary antibody diluted in 2.5% BSA in 0.125% Triton X-100 in PBS. Following washing with 0.125% Triton X-100 in PBS, cells were incubated with a mixture of secondary antibodies conjugated to Alexa Fluor 488, and Alexa Fluor 647 Phalloidin (1:20 in PBS) (for F-actin staining), and DAPI (1:1000 in PBS) for another hour. Reagents were obtained from Cell Signaling Technology (Cambridge, UK). After washing, coverslips were mounted on glass slides and stored at 4 °C in the dark until analysis. The slides were imaged using an LSM 710 Axio Observer confocal laser scanning microscope (Carl Zeiss, Oberkochen, Germany).

### Proteasomal activity

Proteasomal activity was assessed using the Proteasome Activity Fluorometric Assay Kit, (BioVison, CA, USA). Proteasomal inhibitors epoxomycin and MG-132 were from Merck-Millipore (Darmstadt, Germany) and from Enzo Life Sciences (Loerrach, Germany), respectively.

### Electrophysiological recordings

Electrophysiological recordings were performed in hippocampal slices from 2 to 12 months old mice. Slices of 300 μm (whole cell recordings) or 400 μm (field potential recordings) thickness were stored in artificial cerebrospinal fluid (ACSF) gassed with carbogen. Stimulation with biphasic pulses was provided by an A-M systems stimulator (2100, A-M Systems, Sequim, USA). Recording electrodes (3–8 MΩ) were pulled from borosilicate glass (Kimble Chase, Vineland, USA) and all electrophysiological measurements were performed using a HEKA EPC10 patch clamp amplifier and Patchmaster software (HEKA, Göttingen, Germany). Data were filtered and digitized at 3 and 50 kHz, respectively, and stored on hard disk. All experiments were performed at room temperature.

#### Field potential recordings

Dendritic field excitatory postsynaptic potentials (fEPSPs) were recorded from the stratum radiatum of CA1 in response to stimulation of Schaffer collateral axons using glass micropipettes (Kimble Chase Vineland, USA) filled with ACSF. The initial slope of fEPSPs was used as a measure of synaptic response. Stimulation was applied as paired pulses (interval 40–400 ms) at 0.06 Hz. In experiments where we studied LTP fEPSPs were collected at a rate of 0.06 Hz. Baseline values were collected for 10 min (0.06 Hz) prior to LTP which was induced by a 1 s stimulation at 100 Hz.

#### Measurements of miniature excitatory postsynaptic currents (mEPSCs)

Whole-cell voltage-clamp recordings (WCR) from CA1 pyramidal cells were performed with patch pipettes filled with internal Cs-gluconate recording solution containing (in mM): 100 Cs-gluconate, 0.6 EGTA, 5 MgCl_2_, 10 HEPES, 3 ATP, 0.3 GTP, 3 lidocaine N-ethyl bromide, osmolarity: 290 mOsm, pH 7.3. Neurons were voltage-clamped at −70 mV and mEPSCs recorded in 1 s blocks (sampling rate 80 μs). Slices were perfused with ACSF recording solution supplemented with 1 μM tetrodotoxin to prevent action potentials and 100 μM picrotoxin to suppress inhibitory currents^35^. Data were collected for at least 15 minutes.

#### Measurements of release probability

WCR were performed in hippocampal CA1 pyramidal neurons. Excitatory postsynaptic currents (EPSCs) were elicited by stimulation of the CA3 Schaffer collaterals. Patch electrodes were filled with internal Cs-gluconate recording solution. Initial release probability was determined by measuring the rate of block of the NMDA EPSCs by the open channel blocker MK-801 (40 μM) during stimulation at 0.05 Hz^19^,^54^. NMDA EPSCs were pharmacologically isolated using 10 μM NBQX to block AMPA/kainate receptors, 100 μM picrotoxin to block GABA_A_-mediated inhibition and 10 μM CGP55845 to block GABA_B_-mediated inhibition. Holding potential was −40 mV.

After obtaining a stable baseline at 0.05 Hz stimulation MK-801 was washed in. Following a 10 min wash-in period with no stimulus, stimulation was resumed at 0.05 Hz and 80–100 EPSCs were recorded. The release probability of facilitated pulses was assayed in a similar manner. The rate of block was measured in response to 100 Hz 3-pulse trains with a 20 s inter-train interval^35^. The NMDA receptor mediated response was quantified as charge transferred by the EPSCs and normalized to the initial response. A single exponential was fitted to the resulting decay curve. The decay constant (τ) was obtained for each experiment.

#### Data analysis

Analysis of electrophysiological recordings was carried out offline using Patchmaster (HEKA) or IGOR software (Wavemetrics, Lake Oswego, OR, USA). Data fitting and statistical analysis was performed using GraphPad PRISM 5.0 (GraphPad Software Inc., San Diego, CA, USA). Detection of spontaneous mEPSC from records was performed using a threshold above baseline detector^55^ in Neuromatic analysis software (NeuroMatic Version 2.0, http://www.neuromatic.thinkrandom.com). We used a threshold of 3 standard deviations (SD) above the baseline as criterion to detect mEPSCs. For each trace average and SD of the baseline current was analyzed within a 10 ms segment. Since the aim was to characterize the mean properties of mEPSC in control and SPL^fl/fl/Nes^ mice we pooled the mEPSC from all cells in each group. Frequency analysis of mEPSC amplitude and time interval between events was performed. Results were plotted as relative cumulative frequency distribution with equal bin width (n = 100). Comparison of mEPSC frequency and mEPSC amplitude revealed significant differences between control and SPL^fl/fl/Nes^mice (unpaired Student’s *t* test, P < 0.001).

### Electron microscopy

Animals (n = 3 per genotype) were perfused transcardially with 3% glutaraldehyde (Merck, Darmstadt, Germany) in Dulbecco’s PBS (DPBS; Gibco®). Brains were dissected and post-fixed for 4 h at 4 °C in the same fixative washed in DPBS and the hippocampus cut into 100 μm in a vibratome (Leica, Germany). The sections were postfixed in 1% osmium tetroxide in 0.1 M cacodylate buffer (pH 7.4), dehydrated in ethanol, and embedded in Durcupan ACM resin (Fluka Chemicals, Gillingham, UK). Sections were stained en block with 1% uranyl acetate in 70% ethanol. Serial ultrathin (70 or 100 nm) and semithin (2 μm) sections were cut with a diamond knife (Diatome). Ultrathin sections were collected onto formvar-coated copper grids, counterstained with 2% uranyl acetate and lead citrate, and examined in a Tecnai 12 electron microscope (FEI) equipped with 2kx2k TemCam-F224HD camera (TVIPS). Complete series of up to 100 ultrathin sections were used to follow cell morphology and synaptic terminals in 3-dimensions.

#### Quantification of EM data

Images were quantified using Image J software. SV density was calculated as the number of vesicles per square micrometer within regions outlined by 100 nm steps or 300 nm step from the active zone border. SV diameters were measured in 70 nm thick synapse sections. To measure SV diameters clear cut vesicles displaying distinct lipid bilayer were selected. Two measurements between the middle of the bilayer acquired at 90° were acquired for each vesicle and averaged. Number of coated vesicles and endosomal profiles found in nerve terminals were normalized to the length of the active zones acquired from the middle section of synapses. 50 nm thick sections were used to determine the number of docked vesicles in contact with the presynaptic membrane per active zone area.

### Behavioural Analysis

Mice (15–18-month old; n = 9 controls and n = 10 SPL^fl/fl/Nes^) were tested for exploratory locomotion behaviour, spatial learning, short-memory and motor coordination. All experiments were conducted blind with respect to the genotype of the tested animals.

#### Open field

The test apparatus (ActiMot; TSE, Bad Homburg, Germany) was a transparent and infrared light–permeable acrylic test box (45.5 cm × 45.5 cm × 39.5 cm internal measurements). Animals were allowed to freely explore the test arena for 20 minutes. An automated tracking system (Ethovision XT; Noldus, Wageningen, Netherlands) was used to record the total distance travelled by animals.

#### Object placement recognition

After handling and habituation to the empty test arena, mice were subjected to 3 trials of 6-minute training session, during which they were allowed to explore freely 2 identical objects (small glass bottles) that were placed in defined locations of the test arena. Next day, a 6-minute test session was performed, during which the position of one of the objects was changed, while the position of the other remained unaltered. An automated tracking system (Ethovision XT; Noldus) was used to monitor and record the behaviour of the animals. The time the animals spent exploring the object in the novel location and the known location during the test was hand-scored from video recordings.

#### Rotarod

An accelerating rotarod (Bioseb, Vitrolles, France) was used to measure motor coordination, balance, and motor learning abilities. Mice were placed on the rotarod, and the rod rotations were subsequently accelerated from 4 to 40 rpm during the 5-minute trial period. Trials were terminated when animals fell off the rod or when 5 minutes had elapsed, whichever came first. Mice were given 3 trials every day with a 30 min inter-trial interval, for 4 consecutive days.

### Statistical analysis

All values are presented as means ± SEM. Student’s t-test, Mann-Whitney U test and two-way ANOVA were used for statistical analysis of the data. Normality of datasets larger than seven was tested with D’Agostino and Person omnibus normality test. P values lower than 0.05 were considered significant. In the figures asterisks indicate P values as follows: * < 0.05; ** < 0.01; *** < 0.001. The GraphPad Prism 5 software was used for statistical analysis.

## Additional Information

**How to cite this article**: Mitroi, D. N. *et al*. Sphingosine 1-phosphate lyase ablation disrupts presynaptic architecture and function via an ubiquitin- proteasome mediated mechanism. *Sci. Rep.* **6**, 37064; doi: 10.1038/srep37064 (2016).

**Publisher's note:** Springer Nature remains neutral with regard to jurisdictional claims in published maps and institutional affiliations.

## Supplementary Material

Supplementary Figures
