# Supplementary material for: Sphingosine 1-phosphate lyase ablation disrupts presynaptic architecture and function via an ubiquitin- proteasome mediated mechanism
Source: Sci Rep. 2016 Nov 24;6:37064. doi: 10.1038/srep37064 (PMC5121647; doi:10.1038/srep37064)
Supplement: Supplementary Figures [file srep37064-s1.pdf]

Sphingosine 1-phosphate lyase ablation disrupts presynaptic architecture and function via an ubiquitin- proteasome mediated mechanism

Daniel N Mitroi[1*], André Deutschmann[2*], Maren Raucamp[2], Indulekha Karunakaran[1], Konstantine Glebov[3], Michael Hans[2], Jochen Walter[3], Julie Saba[4], Markus Gräler[5], Dan Ehninger[6], Elena Sopova[7], Oleg Shupliakov[7,8], Dieter Swandulla[2], Gerhild van Echten-Deckert[1**]

[1]LIMES Institute, Membrane Biology & Lipid Biochemistry, University of Bonn, Bonn, Germany; [2]Institute of Physiology, University of Bonn, Bonn Germany; [3]Department of Neurology, Molecular Cell Biology Unit, University of Bonn, Bonn Germany; [4]Children's Hospital Oakland Research Institute, University of California, San Francisco, USA; [5]Department of Anaesthesiology and Intensive Care Medicine, Center for Sepsis Control and Care (CSCC), and the Center for Molecular Biomedicine (CMB), University Hospital Jena, Jena, Germany; [6]German Centre for Neurodegenerative Diseases (DZNE), Bonn, Germany; [7]Department of Neuroscience, Karolinska Institutet, 171 77 Stockholm, Sweden; [8]Institute of Translational Biomedicine, St. Petersburg State University, 199034 St. Petersburg, Russia.
*equal contribution, **corresponding author.

Address correspondence to: Gerhild van Echten-Deckert, Kekulé-Institut, Gerhard-Domagk-Str. 1, 53121 Bonn, Germany; phone: +49228732703; fax: +49228734845;
e-mail: g.echten.deckert@uni-bonn.de

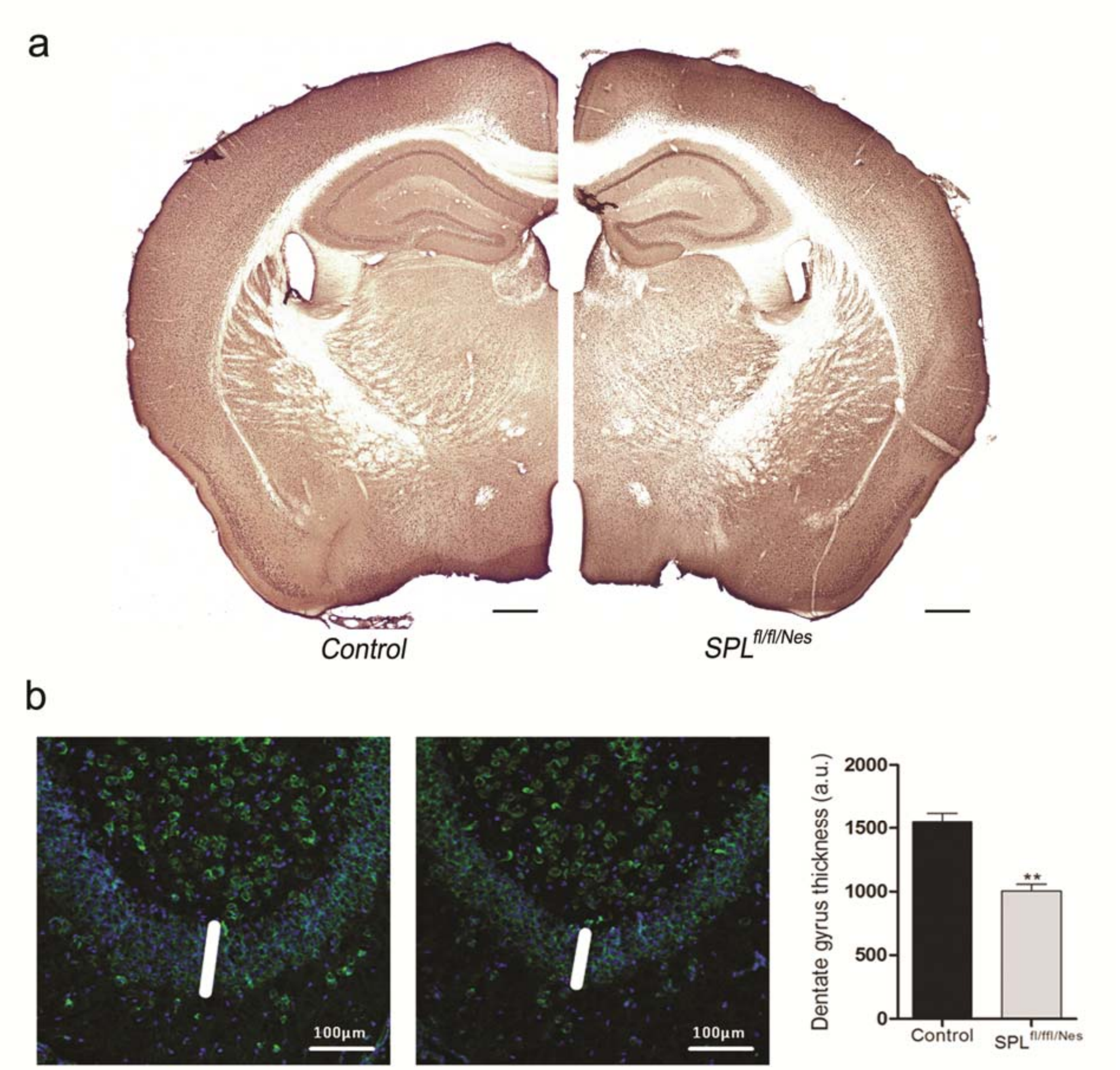

Suppl. Figure 1. The impact of SPL ablation on brain morphology. (**a**) Neuron-specific nuclear protein (NeuN) immunohistochemistry of coronal brain sections from 18-month-old control and SPLfl/fl/Nes mice (4 animals per group). Scale bar, 500 µm. (**b**) Dentate gyrus from 6-month-old control and SPLfl/fl/Nes mice (3 animals per group) stained with DAPI (blue) and Nissl (green) (unpaired t test, P = 0.0014).

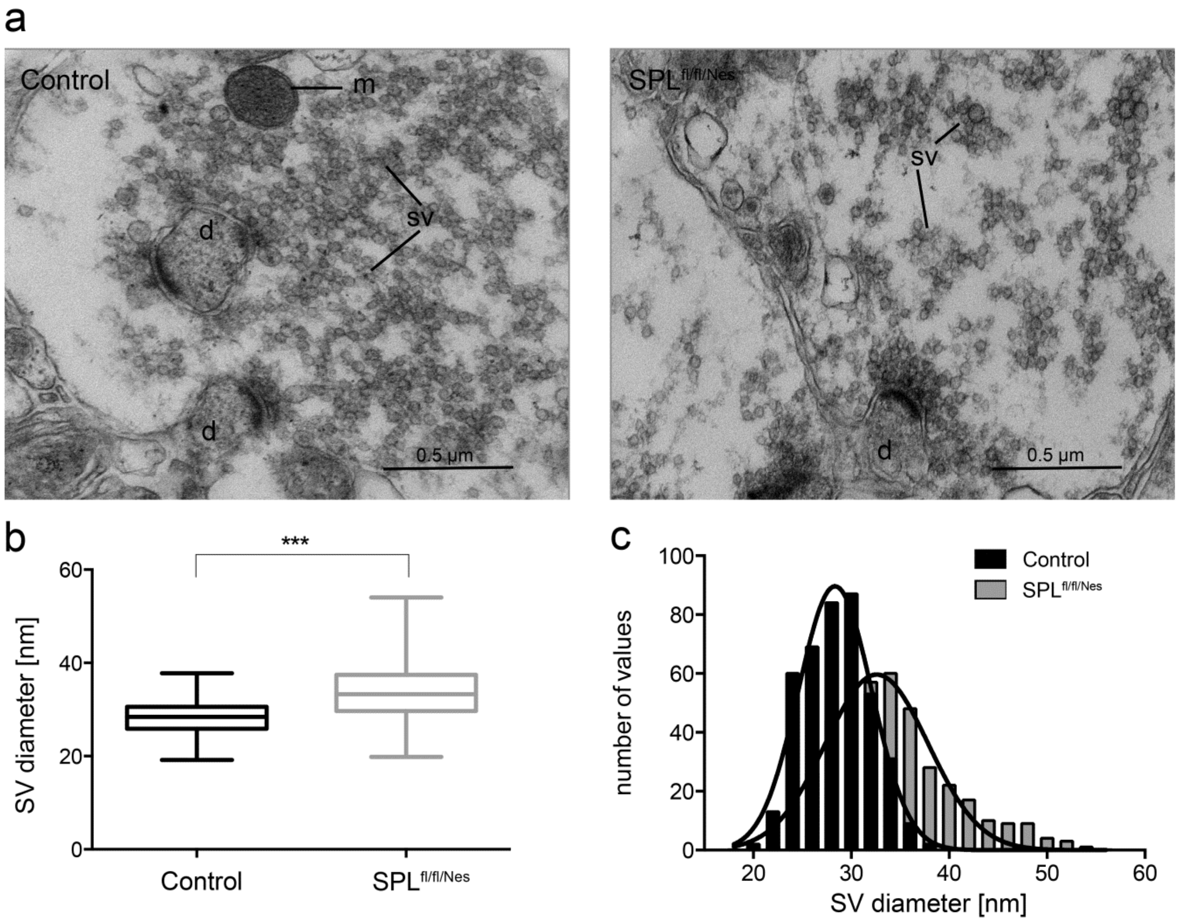

Suppl. Figure 2. Subcellular morphology of giant mossy fiber terminals from CA3 region of murine hippocampus following ablation of SPL. (**a**) EM images of giant mossy fiber boutons from a control and from an $SPL^{fl/fl/Nes}$ hippocampi. (**b**) box and whisker plot illustrating difference in Sv diameters from CA3 giant terminals (n= 410; ***p<0.0001, Student´s t-test). (**c**) frequency distribution of Sv diameters from the same group of nerve terminals. Sv, synaptic vesicles, d, dendritic spines, m, mitochondrion. Thick arrows indicate synaptic contacts.

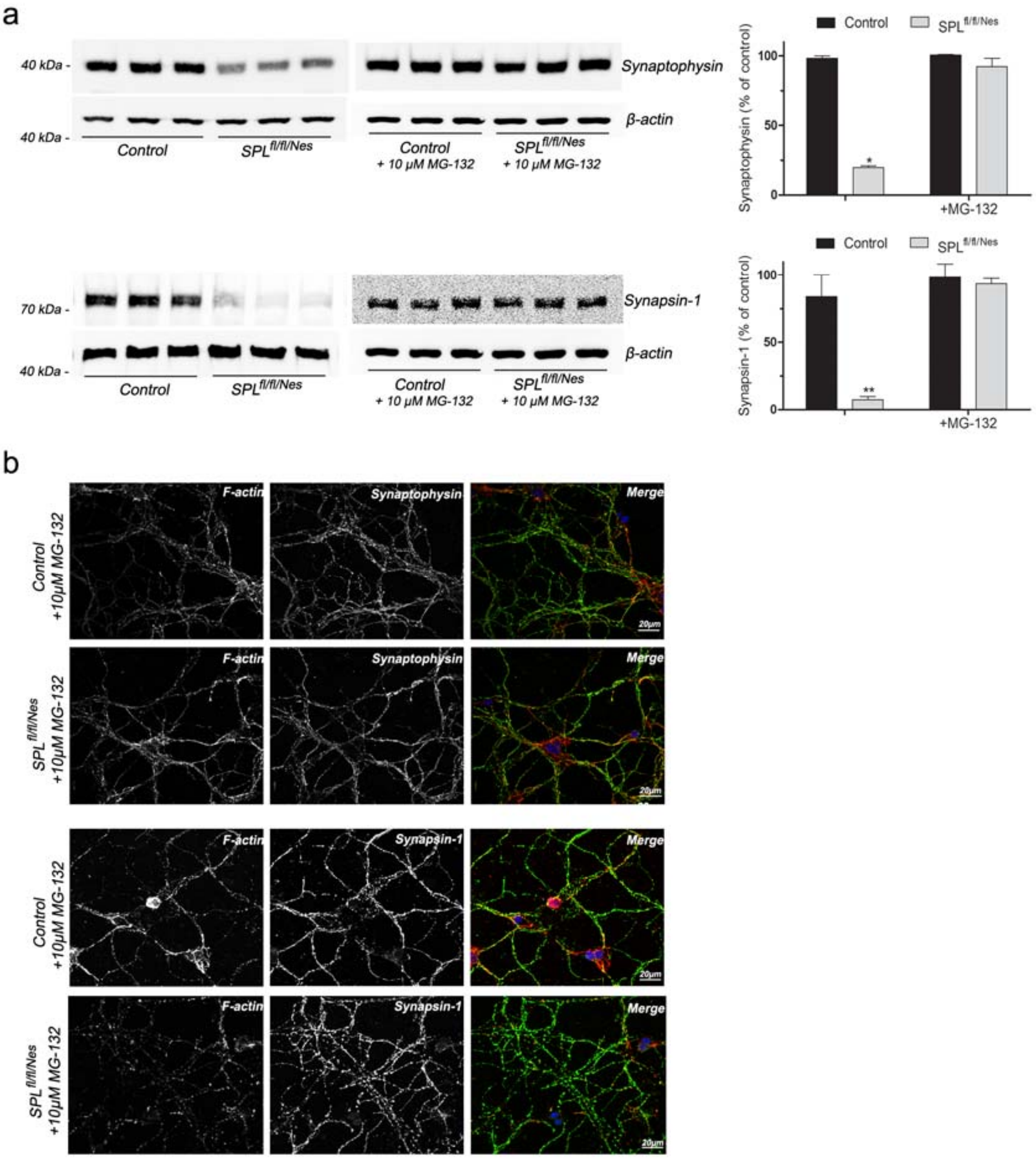

Suppl. Figure 3. **Restoration of pre-synaptic protein expression.** Neuronal cultures generated from cerebella of mice with the indicated genotype were incubated at day 14 in culture with the proteasomal inhibitor MG-132 (10 µM, 24 h, Enzo Life Sciences, Loerrach, Germany). Then cells were harvested and pre-synaptic proteins assessed by (**a**) immunoblotting (two-way ANOVA, $P_{Synaptophysin}$ = 0.012, $P_{Synapsin\text{-}1}$ = 0.0095) and (**b**) immunostaining. F-actin (red), synaptophysin and synapsin-1 (green).

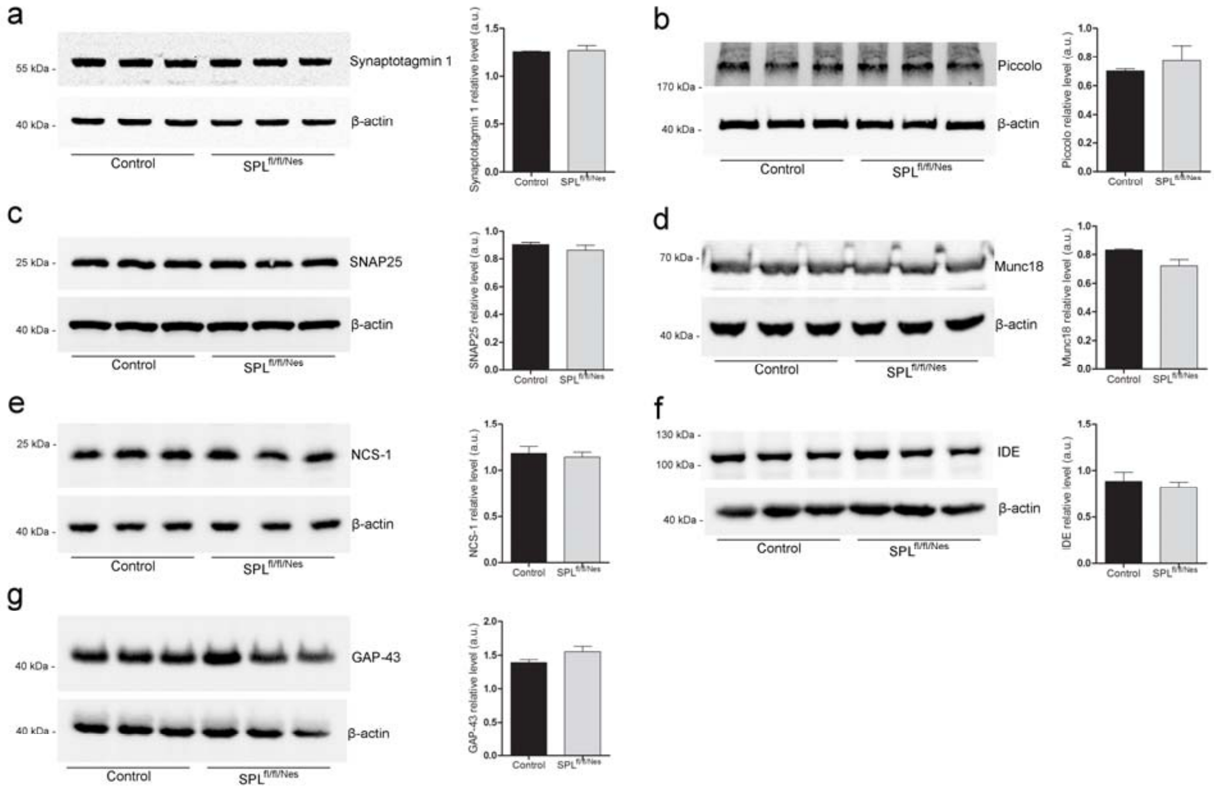

Suppl. Figure 4. **Proteins with unchanged expression in SPL$^{fl/fl/Nes}$ mice.** (**a – g**) Protein amounts were assessed by immunoblotting.
